# The environmental adaptation strategy of seed germination, and roles of the seed pappus on dispersal and hypocotyl hairs on seedling anchorage in *Tamarix ramosissima*

**DOI:** 10.1093/aobpla/plab065

**Published:** 2021-10-09

**Authors:** Caixia Li, Xiaowei Wei, Haiyan Lan

**Affiliations:** Xinjiang Key Laboratory of Biological Resources and Genetic Engineering, College of Life Science and Technology, Xinjiang University, Urumqi, China

**Keywords:** Hypocotyl hairs, seed dispersal, seed germination, seed pappus, *Tamarix ramosissima*

## Abstract

Seed dispersal, germination and seedling establishment are affected by various ecological factors in desert plant species. *Tamarix ramosissima* has evolved multiple strategies to facilitate its survival in harsh environments during the early stages of development. In this study, we investigated the effects of different ecological factors on seed germination and seedling growth, the function of the seed pappus in seed dispersal, as well as the function of the hypocotyl hairs in seedling establishment. We found that the seed germination of *T. ramosissima* was rapid and could occur under a wide range of temperatures (5–30 °C), after long periods of storage (at least 12 months on dispersal), under high concentrations of salts (700–900 mmol·L^−1^) and polyethylene glycol (PEG) 6000 (500 g·L^−1^) and under medium concentrations of alkalis (300–500 mmol·L^−1^). Lower concentrations of salts and PEG promoted seedling growth. The seed pappus had no effect on seed germination, but it might function as an accessory structure that provides a buoyancy force and promotes long-distance seed dispersal. The hypocotyl hairs located on the edge of the hypocotyl end might aid the upright positioning of the seedlings during early development, especially when seed germination occurs under floating or flooding conditions. In conclusion, the germination of *T. ramosissima* seeds and seedling development can occur under diverse types of abiotic stress, and the seed pappus and hypocotyl hairs played an important role in seed dispersal and seedling establishment.

## Teaser

*Tamarix ramosissima* seeds were extremely small and possessed radial pappus on one end. The seed pappus of *T. ramosissima* had no significant effect on seed germination but may provide a buoyancy force that aids the long-distance dispersal of seeds. In addition, we found that the hypocotyl hairs may help the early seedlings become fixed to the surface matrix and provide support to the seedling before the appearance of roots; such a structure might have evolved to facilitate seed dispersal and seedling establishment during flooding and floating water conditions.

## Introduction

Seed germination is the most critical stage for plant survival in extreme circumstances, especially in deserts where drought, large temperature extremes and sandstorms are common (Qu *et al*. 2008). In natural habitats, the soil moisture, salt content, light intensity, temperature regime and sand burial depth significantly affect seed germination (El-Keblawy 2004). Seasonal fluctuations in temperature can determine the germination time of non-dormant seeds, and the effects of such fluctuations can vary among locations (Liu *et al*. 2013). Desert plants can germinate under a wide range of temperatures, which is one of the keys to their ability to cope with harsh environmental conditions (Chen *et al*. 2019). Seed imbibition involves water uptake and is the key first step in early germination (Mandizvo and Odindo 2019). The germination of seeds in deserts often occurs in soils with high salinity and during drought, which reduces the ability of seeds to absorb water and decreases the germination rate and final germination percentage (GP) (Tobe *et al*. 1997). The response to light during germination varies among desert plant species (Flores *et al*. 2015), for example, 21 % of 105 plant species in arid zone of Australia are not able to germinate in the dark without treatment (Jurado and Westoby 1992). Long-term storage at room temperature can also affect seed germination, and storage under such conditions can result in the complete loss of viability of seeds, especially in some desert plants, such as *Salsola* and *Tamarix* (El-Keblawy 2013; Terrones *et al*. 2016).

Desert plants have evolved various strategies to aid their survival in unpredictable environments (Gutterman 2000). Seed dispersal is the means by which plant populations propagate, and it requires less labour compared with transplanting nursery seedlings or shoot cuttings for vegetation rehabilitation (Tobe *et al*. 2001). Generally, seed morphology applies an important effect on seed dispersal patterns (Groenendijk *et al*. 2013). In *Brachyscome ciliaris*, there are two types of seeds: ray seeds, which are smooth and narrow with a minute pappus, and disc seeds, which have a pappus, wing, and curly hairs; disc seeds can disperse a greater distance by wind than ray seeds (Aleman *et al.* 2015). *Centaurea solstitialis* seeds display a task-allocation strategy, wherein large numbers of pappus seeds are produced, which enhances the competitiveness of seedlings through rapid dispersal and seedling establishment; by contrast, non-pappus seeds delay germination and remain dormant, thus ensuring reproduction in changing environments (Florencia Miguel *et al*. 2017).

*Tamarix ramosissima*, commonly known as saltcedar, is a perennial desert halophyte in the family Tamaricaceae that occurs in northwestern China (Zhang and Zhang 2012). It is a small shrub with biseasonal flowering behaviour and a long flowering period; it produces a large number of seeds to ensure its propagation during annual floods, and the combination of these features provides favourable conditions for seedling establishment (Mortenson *et al*. 2011; Yan *et al*. 2011). The seeds of *T. ramosissima* are very small, and there is a symmetrical radial pappus on one end of the seed; such a morphology may have some ecological consequences, such as facilitating seed dispersal through the air or on water (Merkel and Hopkins 1957; Bossard *et al*. 2000). Because of the large deficit between precipitation and potential evapotranspiration in desert areas, *T. ramosissima* seeds are often faced with various types of stress, including drought, high soil salinity, high temperatures and strong light conditions (Shan *et al*. 2008); the effects of various environmental factors on seed germination and seedling growth of this species have not yet been documented. There is thus a need to study the seed germination of *T. ramosissima* under different types of stress, as well as the ecological consequences of seedling establishment. Here, we evaluated (i) the effect of different environmental factors on the seed germination of *T. ramosissima* and (ii) the ecological roles of seed pappus and hypocotyl hairs in *T. ramosissima*. Overall, the results of this study may aid our understanding of the strategies of *T. ramosissima* in adaptation to desert environments in the early stages of development.

## Materials and Methods

### Seed collection

Fully mature seeds of *T. ramosissima* were collected in September 2020 from the natural habitats of a saline–alkaline region at the edge of Gurbantunggut desert, Xinjiang Uygur Autonomous Region, China (44°19′ N, 86°57′ E; 429 mH). Seeds were air-dried at 18–22 °C and 15–20 % relative humidity for 1–2 weeks indoors, and then impurities were removed. The seeds were then sealed in brown paper bags (25 cm × 30 cm) and stored in a wooden cabinet at room temperature or at 4 °C in the refrigerator until experiments.

### Indoor seed dispersal experiment

In an undisturbed room, the intact seeds and de-haired seeds were allowed to fall freely to the ground one by one from heights of 100, 150 and 200 cm (set according to the natural inflorescence position of the plant). Four replicates with 30 seeds (intact or de-haired) of each replicate were applied at each height. The number of seeds within different dispersal radii (10, 20, 30, 40 and 50 cm) were calculated. Taking the releasing point as the centre, the dispersal radius of seeds was defined as 10 cm (within 10 cm), 20 cm (between 10 and 20 cm), 30 cm (between 20 and 30 cm), 40 cm (between 30 and 40 cm) and 50 cm (between 40 and 50 cm).

### Indoor seed falling speed measurement

A device was designed for measuring the seed falling speed (**Supporting Information—Fig. S1**). Three hollow cylinders 10 cm in diameter and 200, 150 or 100 cm in height were prepared; for each cylinder, the upper part consisted of the filter paper, and the lower part was a transparent glass cylinder with a bottom (height: 20 cm, diameter: 10 cm), which was used for observations of falling seeds. For measurements, three cylinders were vertically placed in a room with no airflow, and the intact seeds or de-haired seeds were released from the top centre of the tube with tweezers at different heights. The time was recorded with a stopwatch between two time points from the release of seeds to when seeds touched the bottom of the container. There were four replicates with 30 seeds (intact or de-haired) per replicate for each height. The falling velocity (*v*) was calculated by the following equation: *v* = *h*/*t*, where *h* represents the straight-line height that seeds fell, and *t* represents the time that seeds were airborne.

### Observation of seed and early seedling morphology

A stereomicroscope SMZ25 (Nikon, Japan) was used to observe the morphology of dry seeds, hypocotyl hairs and early seedlings. The seed and seedling indexes were measured using NIS-Elements D4.30 software. The ultra-microstructure morphology of seeds and seedlings (imbibition for 12, 18, 24 and 30 h) was observed using an LEO-1430VP (Carl Zeiss, Germany) scanning electron microscope. Thousand-seed weight was determined by weighing 1000 seeds with a precise analytical balance (accurate to four decimal places). There were four replicates with 1000 seeds per replicate for measurements of 1000-seed weight.

### Cytological observations of early seedling development

The seedlings after germination for 12, 18, 24 and 30 h were harvested and immediately fixed with FAA solution (90 mL of 50 % ethanol, 5 mL of formaldehyde and 5 mL of glacial acetic acid), transferred to a syringe to evacuate the air until all tissues sank to the bottom of the container and finally placed at 4 °C for 24 h. The treated tissues were manipulated according to the following procedure.

#### Dehydration.

 The tissues were immersed in 50 % ethanol and dehydrated twice for 30 and 20 min and then left overnight in 1 % safranine. The stained materials were treated with different concentrations of ethanol: 80 %/1 h, 95 %/1 h, 100 %/1 h and 100 %/40 min.

#### Clearing.

The dehydrated materials were treated with 1/2 absolute ethanol + 1/2 xylene (1 h), xylene (1 h) and xylene (40 min), and a small amount of xylene and fine first-grade paraffin powder were added to the tissue container in a 38 °C incubator overnight.

#### Paraffin inclusion and embedding.

 The incubator temperature was adjusted to 60 °C to completely melt the wax from the last step. The tissues were then transferred to the melted second-grade paraffin for 1 h and immersed in third-grade paraffin at 61 °C (1 h), 62 °C (1 h) and 63 °C (40 min); the paraffin was replaced at each change of the temperature. Finally, the melted paraffin and tissues were carefully poured into a paper tank for embedding.

#### Sectioning.

 The paraffin block containing tissues was trimmed into a small cuboid and cut into 6- to 12-μm slices by a microtome (Leica RM2126).

#### Expanding of section.

The paraffin strips were spread on the surface of warm water and retrieved on the slide, which were then incubated at 37 °C overnight.

#### Deparaffinization.

 Slides with tissues were treated in xylene (two times for 1 h and 5 min) and 1/2 absolute ethanol +1/2 xylene (5 min); they were then transferred to different concentrations of ethanol as: absolute ethanol (5 min), 95 % ethanol (5 min) and 80 % ethanol (5 min).

#### Staining.

 Slides were treated with 1 % safranine for 9–12 h. After 5 min in 80 % ethanol, slides were dipped in 1 % fast green for 10 s, followed by 95 % ethanol (5 min), absolute ethanol (two times, 5 min each), 1/2 absolute ethanol + 1/2 xylene (5 min) and xylene (two times, 5 min each).

#### Mounting.

 A drop of neutral gum was added to the slide, which was covered with a coverslip and sealed with nail polish, and then incubated at 37 °C overnight.

#### Microscopic inspection.

 The images were visualized under a light microscope (Nikon Eclipse NI) and photographed with NIS-Elements software.

### Seed germination experiments

Mature seeds were placed on two layers of filter paper with two layers of absorbent cotton gauze between the paper in 9-cm Petri dishes, which were applied with 8 mL of distilled water or different aqueous solutions. The distilled water or aqueous solution was not replaced until germination was complete. Because the seeds were too small to manipulate, cotton gauze was placed between the filter paper to prevent significant water loss. All Petri dishes were sealed with cling film, placed in an illumination incubator (RXZ-500D-LED; Jiangnan Apparatus Manufactory, China) and subjected to a 16-h light/8-h dark photoperiod (light flux: approx. 100 µmol·m^−2^·s^−1^) at 25 °C and 50 % relative humidity. Germination was recorded when the hypocotyl was observed to protrude from the seed coat. In all germination tests, there were four replicates with 30 seeds per replicate for each treatment. To avoid contamination, Petri dishes were kept sealed during germination, and the GP was recorded every 24 h for a total of 15 days. On the 16th day, the cotyledon width, hypocotyl length and root length of the seedlings were measured using NIS-Elements software; there were three replicates with four seedlings per replicate for seedling growth measurements. The GP was determined by the following equation: GP = *m*/*n* × 100 %, where *m* is the number of germinated seeds, and *n* is the total number of seeds.

#### Removal of the seed pappus.

 To investigate the effect of the seed pappus on germination, the pappus on one end of the seed was carefully removed with a blade, without damaging the integrity of the seed itself.

#### Variable temperature treatments.

 Seeds were subjected to several variable day/night temperature regimes (30/20, 25/15, 20/10 and 15/5 °C) and constant temperature regimes (30, 25, 20, 15, 10 and 5 °C).

#### Different light intensity treatments.

 Seeds in Petri dishes were sealed in transparent coloured (red, yellow, green and blue) paper boxes. Petri dishes under white light or wrapped with foil were used as controls. All Petri dishes were placed under normal light conditions described above. Seed germination was recorded under coloured lights except for the dark treatment, which was performed under green light in a dark room.

#### Salt, alkali and drought treatments.

 Two salts (NaCl and Na_2_SO_4_) and two alkalis (NaHCO_3_ and Na_2_CO_3_) with concentrations of 0, 100, 300, 500, 700 and 900 mmol·L^−1^ and polyethylene glycol (PEG) 6000 (the molecular weight is 6000) at different concentrations (0, 100, 200, 300, 400 and 500 g·L^−1^) were applied during germination to test the performance of seeds under stress.

#### Long storage at room temperature or 4 °C.

 Seeds sealed in brown paper envelopes were stored in a wooden cabinet at room temperature (20–25 °C, 18–25 % relative humidity) or in a refrigerator at 4 °C for approximately 12 months, which were retrieved for seed germination at the beginning of every month from October 2020 to September 2021.

### Statistical analysis

All data were expressed as mean ± SE. One- or two-way ANOVA was used to analyse data collected from different seed germination experiments. If significant main effects were detected, differences were tested by a multiple comparison Tukey’s test at the 0.05, 0.01 and 0.0001 significance thresholds. Statistical analyses were carried out using SPSS 22 software; graphs were prepared using GraphPad Prism version 5.0 software.

## Results

### Characteristics of the seed morphology of *T. ramosissima*

**Figure 1.**
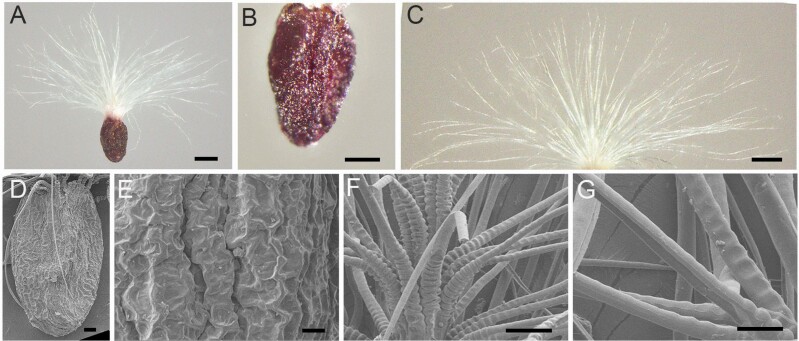
The morphology and micro-structure of *T. ramosissima* seed. (A–C) Seed under stereomicroscope; (D–G) seed under scanning electron microscope. (A) Dry intact seed; (B) seed without pappus; (C) seed pappus; (D) seed without pappus; (E) seed coat surface; (F) the joint part of the pappus connected with seed; (G) the upper part of the pappus. The scale bar in (A, C) is 200 μm, in (B) is 100 μm, in (D, G) is 50 μm and in (E, F) is 20 μm.

Mature dry seeds of *T. ramosissima* were in the shape of date pits and dark brown in colour; a large number of long radial hairs (pappus) were present at one end (Fig. 1A–C). Seeds without pappus were 365.43 μm long and 249.72 μm wide, and the 1000-seed weight was approximately 38 mg (Table 1). Scanning electron microscopy revealed that the seed coat surface had irregular rough ridges (Fig. 1D and E). On one end of the seed coat, a cluster of white, long and soft pappus protruded, and these hairs were longer than the seeds and usually spread out radially when being dispersed (Fig. 1C). A string of bead-like structures was present on the root of each hair, with a smooth extension from the middle toward the upper part (Fig. 1F and G).

**Table 1.** Characteristics of *T. ramosissima* seeds. Values are means ± SE of four replicates.

Seed type	1000-seed weight (mg)	Length (μm)	Width (μm)
Intact seed	38.25 ± 0.95	–	–
Seed without pappus	28.75 ± 1.44	365.43 ± 15.64	249.72 ± 21.35
Seed pappus	8.75 ± 0.48	1011.93 ± 52.45	–

### Function of the seed pappus

**Figure 2.**
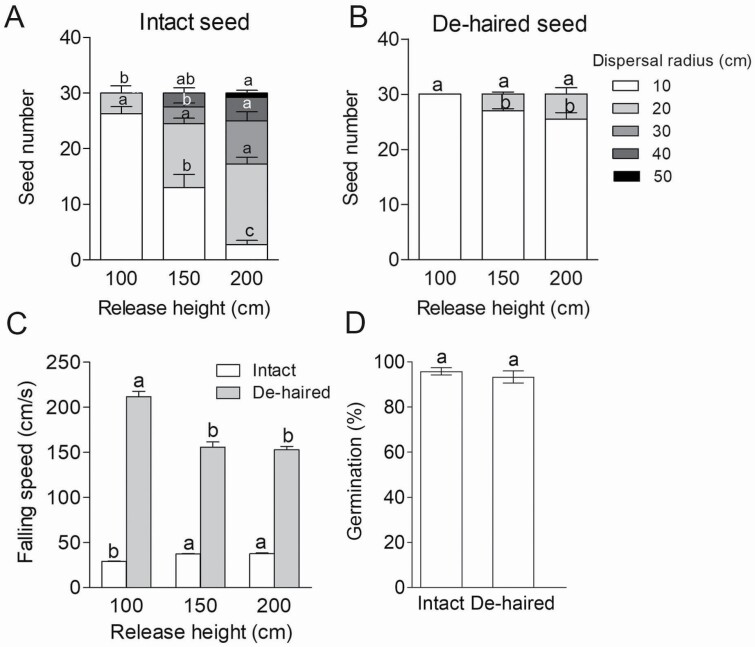
The effect of seed pappus on seed dispersal and germination. (A) Dispersal test of intact seeds indoors; (B) dispersal test of de-haired seeds indoors; (C) the falling speed of intact and de-haired seeds; (D) the GP of intact and de-haired seeds. In (A) and (B), different lowercase letters indicate significant difference (*P* < 0.05) of the seed number between different release heights within the same dispersal radius; in (C), different lowercase letters indicate significant difference (*P* < 0.05) of the falling speed between different release heights within the same seed type; in (D), the same lowercase letter represents no significant difference (*P* < 0.05) of the GP between intact and de-haired seeds. Values are means ± SE of four replicates.

To investigate the effects of seed pappus on germination and dispersal, the seed pappus was removed from the seed by cutting the roots of the hairs without damaging the seed coat and the embryo (referred to as de-haired seeds). The intact and de-haired seeds were released from different heights. The dispersal area increased as the release height increased. When the release height was less than 100 cm, most of the intact seeds fell down on the ground within 10 cm from the release point; when the height increased to 200 cm, the number of seeds falling within a 10-cm area was significantly reduced, and most seeds fell between 20 and 40 cm (Fig. 2A). When the de-haired seeds were released from different heights, most of them fell within 10 cm (Fig. 2B), and the speed at which the de-haired seeds fell was significantly faster compared with intact seeds (Fig. 2C), suggesting that the seed pappus plays an important role in the dispersal of *T. ramosissima* seeds. Removal of the seed pappus had no significant effect on germination (Fig. 2D). These results suggest that the seed pappus is an accessory structure with the main function of aiding seed dispersal in *T. ramosissima.*

### Morphological and structural characteristics of seedlings during early seed germination

**Figure 3.**
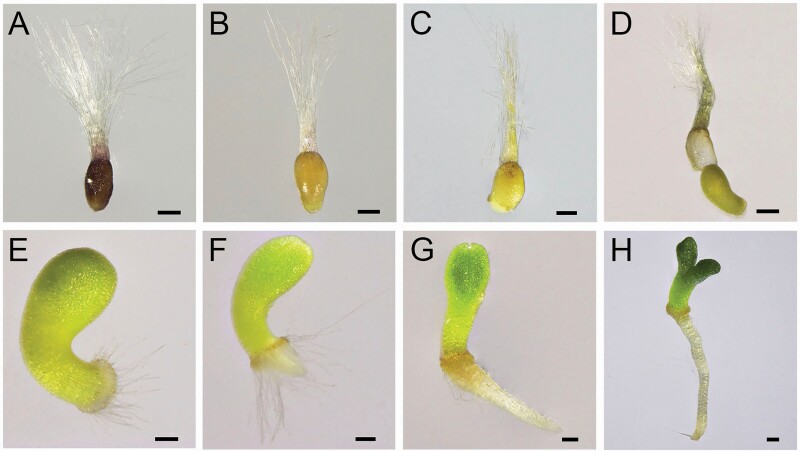
The morphological and structural changes of seeds in early germination of *T. ramosissima*. (A–H) The seed/seedling morphology at 1, 3, 12, 24, 34, 48, 72 and 120 h after the imbibition. The scale bar in (A–D) is 200 μm and in (E–H) is 100 μm.

Continuous observations of the initiation period of the germination of intact seeds were made, and morphological and structural changes were characterized. Four stages were noted. The first stage was at approximately 3 h after imbibition; the seed coat extended from its original shrunken condition as a light-coloured and semi-transparent capsule, and the hypocotyl was in the process of breaking through the seed coat (Fig. 3A and B). The second stage was at approximately 12–24 h after imbibition; the cotyledons were enlarged, the hypocotyl was further extended and the embryo was separated from the seed coat (Fig. 3C and D). A ring-like structure and the initial form of the hairs appeared on the end of the hypocotyl (Fig. 3E). The third stage was between 34 and 48 h after imbibition; the hypocotyl hairs were further elongated from approximately 16 to 740 μm, and the radicle protruded from the end of the hypocotyl (Fig. 3F). The fourth stage was at approximately 72–120 h after imbibition; the hypocotyl hairs gradually shrunk away from the root collar, the radicle quickly elongated and the two cotyledons separated and became dark green (Fig. 3G and H).

### Effects of temperature, light quality and storage time on seed germination

**Figure 4.**
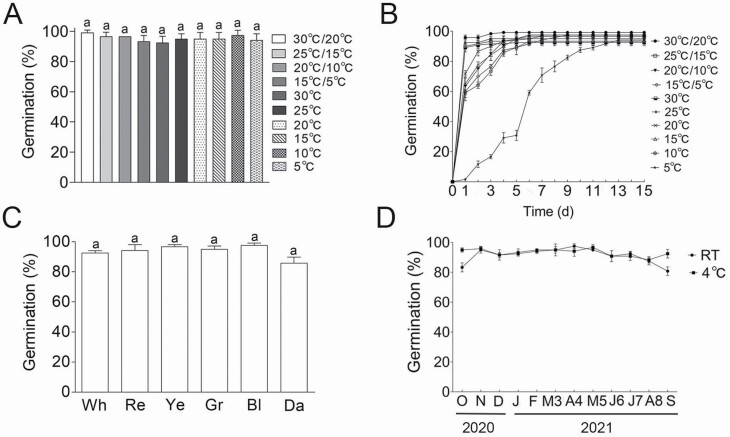
Effects of different conditions on seed germination of *T. ramosissima.* (A, B) temperature; (C) light quality. Wh, Re, Ye, Gr, Bl and Da represent the white, red, yellow, green, blue and dark light, respectively; (D) the storage time. J: January; F: February; M3: March; A4: April; M5: May; J6: June; J7: July; A8: August; S: September; O: October; N: November; D: December. RT: room temperature. 2020 and 2021 indicate the years of seed germination. In (A) and (C), the same lowercase letter indicates no significant difference (*P* < 0.05) of the GP between different temperatures or light qualities. Values are means ± SE of four replicates.

There was no significant difference in the final GP under constant or fluctuating temperature conditions. The germination rate was significantly reduced under lower temperature (5 °C). Only a slight delay in germination was observed during the first couple of days at temperatures from 10 to 20 °C, and the GP was highest from 25 to 30 °C. The germination rate was slightly increased at higher temperatures (i.e. greater than 20 °C) (Fig. 4A and B). Our results indicate that *T. ramosissima* can germinate at a wide range of temperatures. Light quality had no significant effect on seed germination (Fig. 4C); however, darkness slightly reduced the GP, indicating that light may not be essential for the seed germination of *T. ramosissima*. To analyse the effects of storage time and conditions on seed germination, the monthly GP was determined. As the storage time extended (a total of 12 months from the harvest) at different temperatures, the GP did not significantly change, and no differences were noted between storage at 4 °C and room temperature storage (Fig. 4D).

### Effects of salt, alkali and drought stress on seed germination and seedling growth

#### Seed germination.

**Figure 5.**
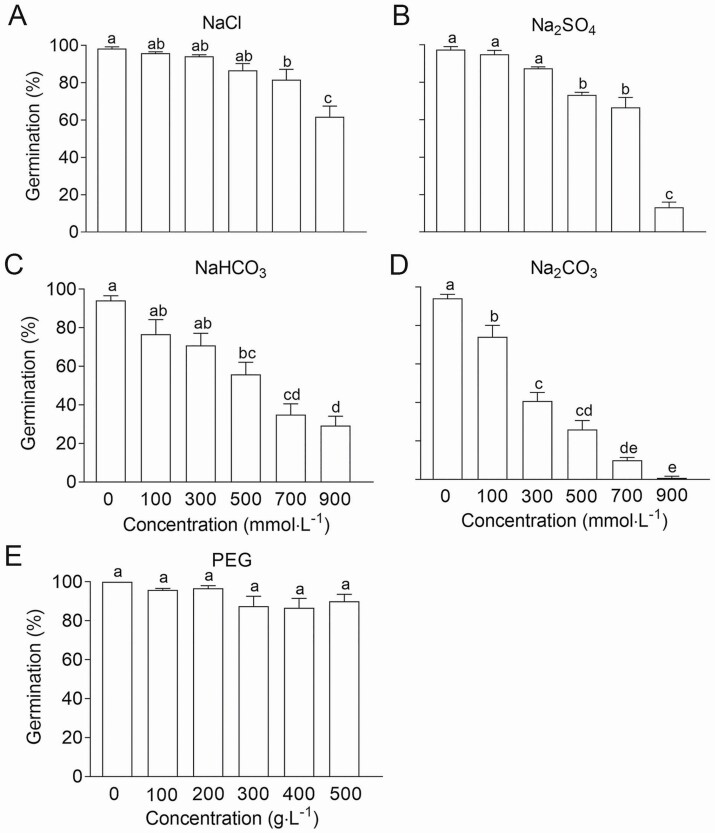
Effects of salts, alkalis and PEG stress on seed germination of *T. ramosissima*. (A) NaCl (0, 100, 300, 500, 700, 900 mmol·L^−1^); (B) Na_2_SO_4_ (0, 100, 300, 500, 700, 900 mmol·L^−1^); (C) NaHCO_3_ (0, 100, 300, 500, 700, 900 mmol·L^−1^); (D) Na_2_CO_3_ (0, 100, 300, 500, 700, 900 mmol·L^−1^); (E) PEG 6000 (0, 100, 200, 300, 400, 500 g·L^−1^). Different lowercase letters indicate significant difference (*P* < 0.05) of the GP between different concentrations of salts or alkalis or PEG. Values are means ± SE of four replicates.

 NaCl and Na_2_SO_4_ (salts), NaHCO_3_ and Na_2_CO_3_ (alkalis), and PEG 6000 (drought) treatments were applied during seed germination. The GP was significantly reduced at higher salt concentrations (≥700 mmol·L^−1^ for NaCl; ≥500 mmol·L^−1^ for Na_2_SO_4_). The GP decreased more rapidly as the concentration of Na_2_SO_4_ increased compared with NaCl. The GP was reduced by approximately 60 and 10 % under 900 mmol·L^−1^ NaCl and Na_2_SO_4_, respectively (Fig. 5A and B). Alkalis more strongly inhibited germination compared with salts when applied at the same concentration. NaHCO_3_ and Na_2_CO_3_ significantly reduced the GP at 500 mmol·L^−1^ and 100 mmol·L^−1^ respectively, and the GP was reduced to 30 and 0.83 % at 900 mmol·L^−1^ of NaHCO_3_ and Na_2_CO_3_, respectively (Fig. 5C and D). Our results suggest that divalent salts and alkalis may have stronger inhibitory effects on the germination of *T. ramosissima* seeds. In the PEG 6000 treatment, no significant changes in the final GP were observed as the concentration of PEG 6000 increased, suggesting that germinating *T. ramosissima* seeds can tolerate drought stress (Fig. 5E). Two-way ANOVA showed that salt type, salt concentration and their interaction, as well as alkali type, alkali concentration and their interaction all significantly affected seed germination (*P* < 0.0001) (Tables 2 and 3).

**Table 2.** The two-way ANOVA of effects of salt type, salt concentration and their interaction on seed germination of *T. ramosissima*

Independent variable	df	*F-*value	*P-*value
Salt type	1	57.8	<0.0001
Salt concentration	5	96.516	<0.0001
Salt type × salt concentration	5	15.173	<0.0001

**Table 3.** The two-way ANOVA of effects of alkali type, alkali concentration and their interaction on seed germination of *T. ramosissima*

Independent variable	df	*F-*value	*P-*value
Alkali type	1	7.843	<0.0001
Alkali concentration	5	45.025	<0.0001
Alkali type × alkali concentration	5	0.934	<0.0001

#### Seedling growth.

**Figure 6.**
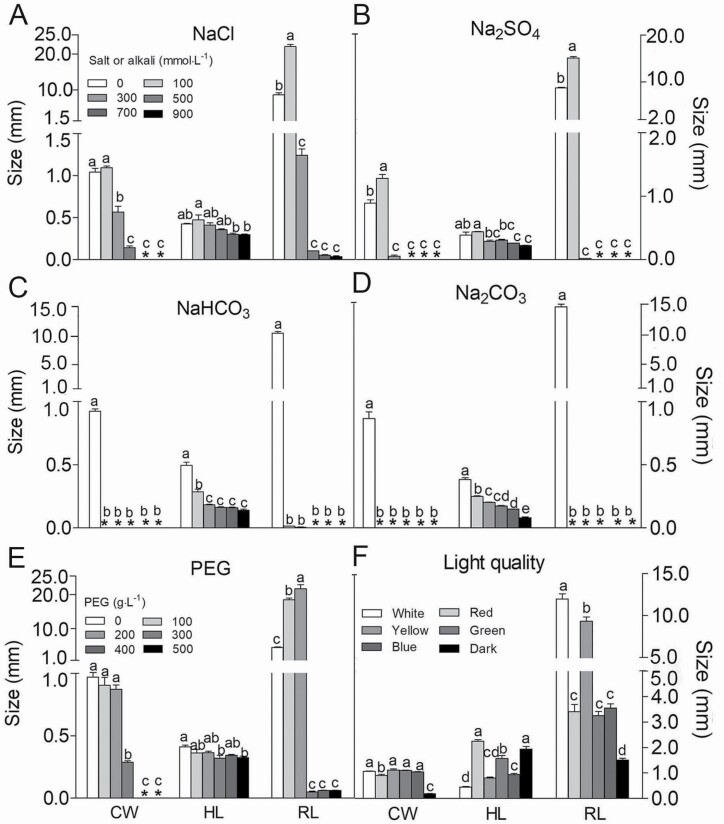
Effects of different stresses on seedling growth of *T. ramosissima*. (A–F) The cotyledon width, hypocotyl length and root length under NaCl, Na_2_SO_4_, NaHCO_3_, Na_2_CO_3,_ PEG 6000 and light quality treatments. CW: cotyledon width; HL: hypocotyl length; RL: root length. The legend of (B–D) is the same as (A). *The value of zero. Different lowercase letters indicate significant differences between different concentrations of salts, alkalis and PEG (or different light qualities) of cotyledon width or hypocotyl length or root length (*P* < 0.05). Values are means ± SE of four replicates.

 Clear differences in seedling growth under salt, alkali and drought stress were observed. Compared with water treatment, lower concentrations (≤100 mmol·L^−1^) of NaCl and Na_2_SO_4_ significantly promoted radicle growth; however, cotyledon and radicle development was significantly inhibited as the concentration of NaCl and Na_2_SO_4_ applied increased (NaCl≥700 mmol·L^−1^, Na_2_SO_4_≥500 mmol·L^−1^) (Fig. 6A and B). Alkalis more strongly inhibited seedling growth compared with salts; 100 mmol·L^−1^ NaHCO_3_ and Na_2_CO_3_ significantly inhibited seedling growth, as no expansion of the cotyledons was observed (Fig. 6C and D). Lower PEG 6000 concentrations (≤200 g·L^−1^) could promote radicle elongation, and cotyledon expansion was significantly inhibited when the PEG 6000 concentration exceeded 400 g·L^−1^ (Fig. 6E; **Supporting Information—Fig. S2**). The radicle was shorter and the hypocotyl was longer under red, yellow, green and blue light compared with white light, which indicates that light is important for the establishment of *T. ramosissima* seedlings (Fig. 6F).

### The micro-structure of hypocotyl hairs and their function

**Figure 7.**
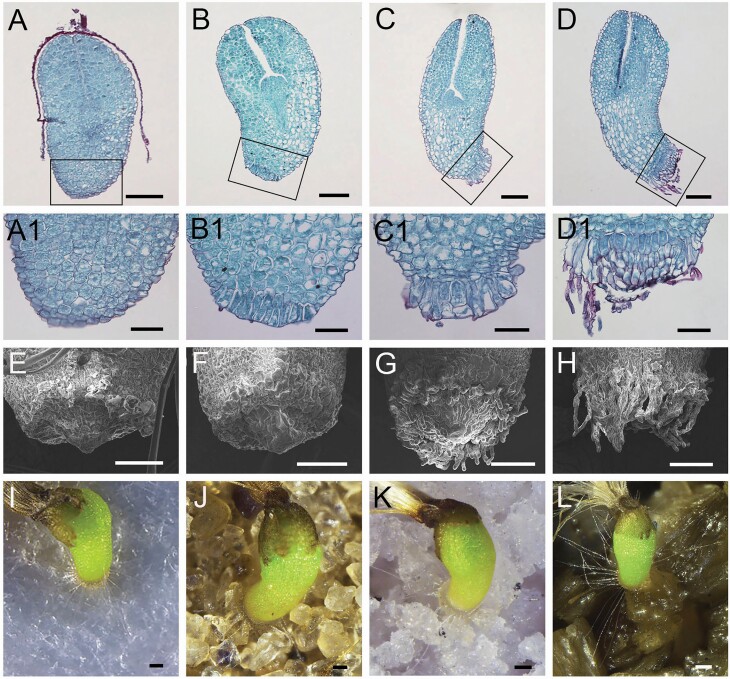
Observations on the developmental structure of hypocotyl hairs of *T. ramosissima.* (A–D) Micro-structure at 12, 18, 24 and 30 h after imbibition; (A1–D1) the enlarged micro-structure corresponding to (A–D); (E–H) the ultra-microstructure under the scanning electron microscope corresponding to (A–D); (I, J) seeds germinated on different substrates. (I) filter paper; (J) fine sands; (K) ground perlite; (L) vermiculite. The scale bar in (A–D) and (I–L) is 100 μm and in (A1–H) is 50 μm.

In the early stage of seed germination, we observed that a ring of apparently elongated cells was present on the bottom edge of the hypocotyl (Fig. 7A and B, A1 and B1). As the hypocotyl extended, a root collar structure was formed from these long cells (Fig. 7C and C1); subsequently, many long, thin hairs were generated from the root collar structure on the periphery of the hypocotyl (Fig. 7D and D1). Scanning electron microscopy revealed an elongated cell layer on the edge of the bottom of the hypocotyl as well as the initiation of the root projection (Fig. 7E and F). As the seedlings developed, the hypocotyl hairs were elongated and cylindrical in shape and had spherical tips (Fig. 7G and H). To investigate the function of hypocotyl hairs, seeds were sown on different substrates (filter paper, fine sands, ground perlite and vermiculite). We found that the hypocotyl hairs were radially spread out on the ground of the solid matrixes around the edge of the root collar, which may provide support for the establishment of seedlings early during germination. When the matrix had a condensed solid surface or presented as fine particles, the hypocotyl hairs may be in close contact with the matrix surface (Fig. 7I and J), but when intervening gaps between the particles are present, the radial hypocotyl hairs may interact with the nearest support to help fix the seedlings on the matrix surface (Fig. 7K and L). Before the elongation of the roots and the extension of the cotyledons, the hypocotyl hairs may help the seedlings become fixed on the matrix surface, which aids their subsequent development.

## Discussion

*Tamarix ramosissima* is a dominant species in desert and semi-desert areas with excellent windbreak and sand fixation properties; seed dispersal is the mechanism by which *T. ramosissima* propagates in its natural habitat (Brock 1994). *T. ramosissima* seeds are extremely small and have hairy appendages (Zhang *et al*. 2002), which are considered adaptations for survival in adverse environments (Wang *et al*. 2019). Few studies have examined the various factors affecting seed germination and seedling growth in *T. ramosissima.* In this study, we investigated the morphology and structure of seeds and seedlings in the early germination period, the seed germination characteristics and early seedling growth behaviours under different types of stress and the ecological roles of seed pappus (hairs) and hypocotyl hairs in *T. ramosissima*. We found that the seed germination of *T. ramosissima* was rapid and could occur under a wide range of temperatures and under salinity, alkali and drought stress. The seed pappus had a significant effect on seed dispersal but no effect on germination. Hypocotyl hairs produced on the lower part end of the hypocotyl might aid the fixation of early seedlings on solid surfaces to promote further growth. All these properties aid the ability of *T. ramosissima* to survive in harsh desert environments. Our findings contribute to our understanding of the strategies desert species have evolved to enhance their survival in harsh environments during the early stages of seedling development.

Seed morphological characteristics are considered as an adaptation for reproduction and dispersal (Andersen 1993). Various types of seed accessory structures have been identified in desert species, such as wings/bracts, pappus/hairs, hooks/spines and awns. Typical halophytes in *Atriplex*, *Anabasis*, *Halogeton*, *Tamarix* and *Salsola* can produce wind-borne seeds with flat-winged or pappus-like seed appendages, which can also increase their dispersal distance on the water surface with sufficient rainfall (Liu *et al*. 2014). Our data showed that the seeds of *T. ramosissima* were extremely small and possessed radial pappus on one end, which did not significantly affect seed germination. Generally, this appendage may supply a buoyancy force that helps seeds float in the air or on the water to disperse larger distances (Horton and Clark 2001; Jongejans and Merkel 2001), which increases the likelihood that the seeds will colonize locations suitable for seedling establishment (Natale and Reinoso 2016). Our tests showed that the seed pappus can help seeds disperse larger distance, and the dispersal distance was significantly extended as the height at which seeds were released increased, which is consistent with previous studies indicating that the main determinants of dispersal distance in seeds include variables such as the height of seed maturation and wind speed (Muller-Landau *et al*. 2008; Thomson *et al*. 2011). Our results suggest that the seed pappus may aid seed dispersal in *T. ramosissima.*

Seed germination is affected by various environmental factors, especially in desert plant species (Gutterman 2000). Temperature and light are the most important ecological factors regulating seed germination when water is available (Benvenuti *et al*. 2001; Gairola *et al*. 2011). Most desert halophytes can germinate under a wide range of temperatures (Al-Hawija *et al*. 2012), and species vary in their germination patterns under different temperatures. In some species such as *Triglochin maritima* and *Haloxylon recurvum*, light is essential; others are light-insensitive, such as *Suaeda fruticosa* and *Atriplex griffithii* (Khan and Ungar 1997, Khan and Gulzar 2003). In this study, we found that *T. ramosissima* seeds could germinate under a wide range of temperatures and were light-insensitive, which may be advantageous for survival in extreme desert environments. The storage time is important for the persistence of seeds in the seed bank in natural habitats. The factors affecting seed longevity during storage include temperature, seed properties and water content. In general, 4 °C is an ideal temperature for seed storage (Pradhan and Badola 2012). In this study, the GP of *T. ramosissima* seeds was high at both 4 °C and room temperature within 12 months from October (seeds harvested from natural dried inflorescence in September) to the following September. It has been reported that *T. ramosissima* seeds can remain viable in laboratory for more than 40 weeks and more than 4 months in winter in ideal seed bank conditions; however, they can only remain viable for 45 days in summer (Jacobs and Sing 2007).

For many desert plants, the major ecological factors restricting germination include salinity, alkali and drought stress (Sidari *et al*. 2008). In this study, higher concentrations of salts (≥700 mmol·L^−1^ for NaCl; ≥500 mmol·L^−1^ for Na_2_SO_4_) or alkalis (≥500 mmol·L^−1^ for NaHCO_3_; ≥100 mmol·L^−1^ for Na_2_CO_3_) significantly inhibited the germination of *T. ramosissima* seeds. The mechanisms by which salt and alkali stress affect ionic equilibrium differ (Yang *et al*. 2007): neutral salts mainly disrupt the ions and osmotic balance, whereas alkali stress also inhibits the absorption of mineral elements (Guo *et al*. 2020). The increase in pH associated with alkaline salts can also affect germination. Generally, the inhibition of alkali stress is stronger than that of salt stress when salts and alkalis are applied at the same concentration, which may be caused by the interaction between sodium ions and high pH (Lin *et al*. 2011). Compared with other glycophytes or desert plant species, *T. ramosissima* was much more tolerant of salts or alkalis during seed germination. The final GP of perennial ryegrass seeds decrease significantly when the concentration of NaCl or Na_2_CO_3_ is greater than 200 mmol·L^−1^ or 50 mmol·L^−1^, respectively (Lin *et al*. 2018). Germination of another desert species in Tamaricaceae, *Reaumuria soongorica*, is almost completely lost when it is treated with 300 mmol·L^−1^ of mixed salts (NaCl: Na_2_SO_4_ = 1:1) (Wang *et al*. 2017). Plant species vary in their degree of drought tolerance during seed germination. Tomato (non-halophyte) is highly sensitive to drought stress, as 8 % PEG treatment can significantly reduce the germination rate of 15 different varieties (Jokanović and Zdravković 2015). The desert plant *Eremosparton songoricum* can hardly tolerate 15 % PEG during germination (Li *et al*. 2013). Our study showed that *T. ramosissima* seeds could germinate under much higher PEG concentrations (at least 30 %). *T. ramosissima* is common in desert areas with high evaporation and low rainfall (Wang *et al*. 2011). Saline–alkaline and drought stresses are major determinants of seedling establishment. Our data suggest that higher concentrations of salts/alkalis or PEG 6000 significantly reduced germination, whereas lower concentrations (with the exception of alkalis) could promote the growth of *T. ramosissima* seedlings. In several plants, a certain range of salt concentrations can act as a source of nutrients that promotes plant growth (Yi *et al*. 2007; Cao *et al*. 2015; Sun *et al*. 2019); alkali stress typically inhibits seedling growth (Li *et al*. 2010). This was also the case for *T. ramosissima* in our study.

In aquatic plants in the families Helobiae, Nymphaeaceae and Potamogetonaceae, early seedlings commonly have a ring of hairs on the lower end of the hypocotyl immediately after germination (Haines and Lye 1975; Churchill 1983); this has also been reported in 12 terrestrial plant species (Morita *et al*. 1995) but has not yet been documented in desert plants. The main function of hypocotyl hairs is to provide support and aid the fixation of seedlings to the surface matrix in early development (Robinson *et al*. 2008). In our study of the early seedlings of *T. ramosissima*, hairs were present at the edge of the hypocotyl after imbibition for 24 h and shrunk as the roots extended. Further observation revealed that the hypocotyl hairs permit the seedlings to be positioned upright, which allows *T. ramosissima* seeds to germinate in flooding or floating water (Birken and Cooper 2006); thus, hypocotyl hairs in seedlings might aid their fixation to solid surfaces in water. In some species, there is mucus in hypocotyl hairs, which can help hypocotyl hairs adhere to the substrate. The hypocotyl hairs can also regulate the water absorption of seedlings (Young and Martens 1991; Aronne and Micco 2004). However, no mucilage on the hypocotyl hairs of *T. ramosissima* was detected.

## Conclusions

**Figure 8.**
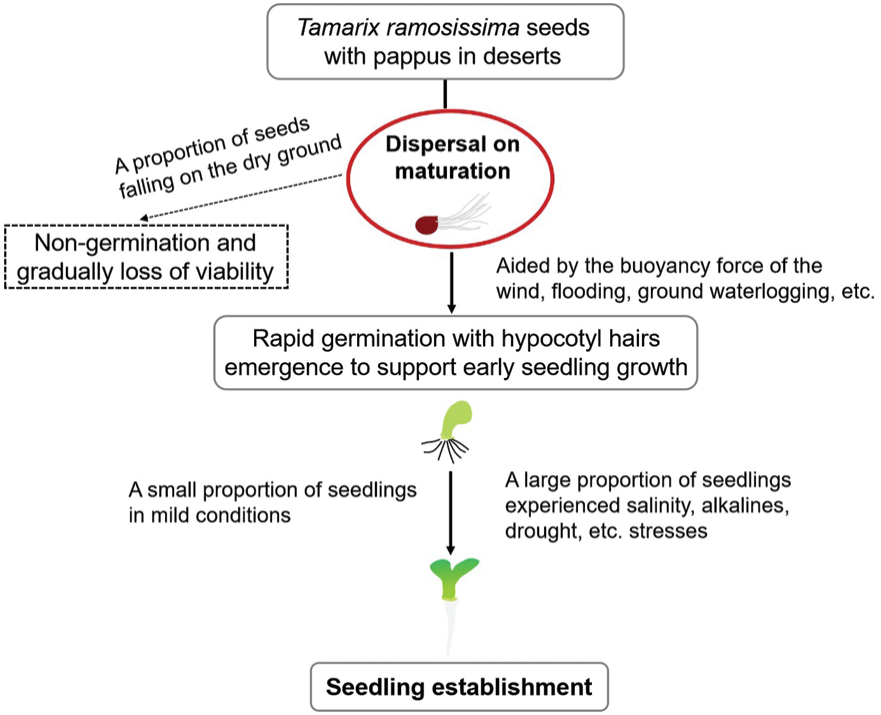
The proposed model of the seed dispersal of *T. ramosissima.*

In this study, we investigated the effect of various environmental factors on the seed germination of *T. ramosissima* and the functions of the seed pappus and hypocotyl hairs. Our data showed that the seeds of *T. ramosissima* could germinate quickly under a wide range of temperatures; germinating seeds could tolerate high concentrations of salts, alkalis and PEG stress, and lower concentrations of these substances could promote seedling growth; and compared with salt and drought stress, alkalis more strongly inhibited the seed germination and seedling growth of *T. ramosissima*. The seed pappus of *T. ramosissima* had no significant effect on seed germination but may provide a buoyancy (or wind) force that aids the long-distance dispersal of seeds. In addition, we found that the hypocotyl hairs on early seedlings may help the seedlings become fixed to the surface matrix and provide support to the seedling before the appearance of roots; such a structure might have evolved to facilitate seed dispersal and seedling establishment during flooding and floating water conditions. Based on our data, we proposed a model for seed dispersal and seedling establishment of *T. ramosissima* in natural habitats (Fig. 8). Our model describes the seed dispersal strategy, seed germination characteristics and the possible mechanism of seedling establishment of *T. ramosissima* in desert environments. Our findings provide new insight into the survival strategy of *T. ramosissima* and similar species in extreme desert environments in early developmental stages.

## Supporting Information

The following additional information is available in the online version of this article—

**Figure S1.** The schematic diagram of the device for seed falling speed test.

**Figure S2.** The morphology of seedlings under different concentrations of PEG in germination.

plab065_suppl_Supplementary_Figures

plab065_suppl_Supplementary_Data

## Data Availability

The raw data used in this study are also available as Supporting Information.

## Notes

**XXXX. Chief Editor**: Tom Buckley
