# Supplementary material for: The environmental adaptation strategy of seed germination, and roles of the seed pappus on dispersal and hypocotyl hairs on seedling anchorage in Tamarix ramosissima
Source: AoB Plants. 2021 Oct 9;13(6):plab065. doi: 10.1093/aobpla/plab065 (PMC8600553; doi:10.1093/aobpla/plab065)
Supplement: plab065_suppl_Supplementary_Figures [file plab065_suppl_supplementary_figures.pdf]

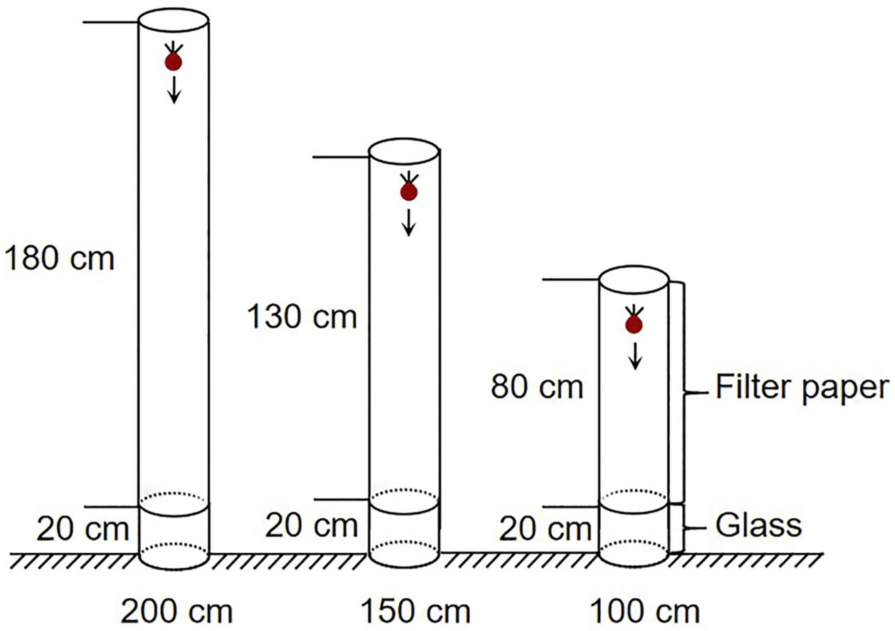

Figure S1. The schematic diagram of the device for seed falling speed test.

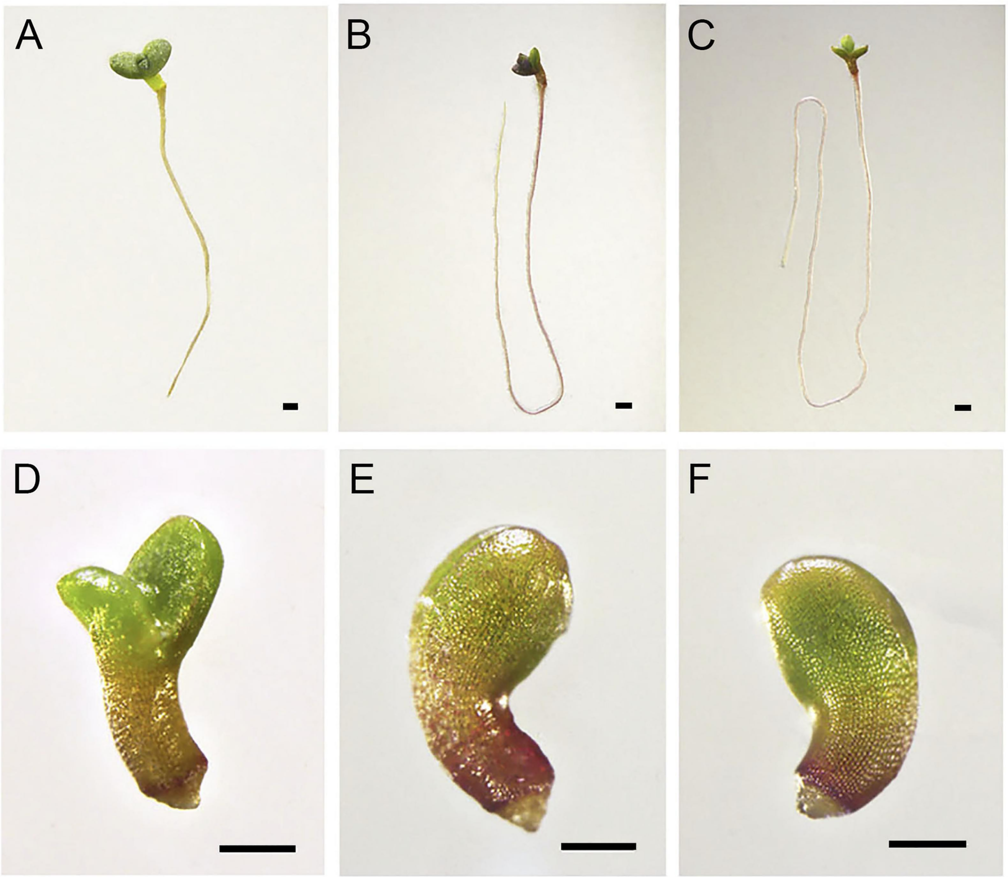

Figure S2. The morphology of seedlings under different concentrations of PEG in germination. A-F: The sixteen-day seedlings after germination in PEG 6000 solution of 0, 100, 200, 300, 400, 500 $g{\cdot}L^{-1}$. The scale bar in A-F is 200 μm.
